# Explorative Combined Lipid and Transcriptomic Profiling of Substantia Nigra and Putamen in Parkinson’s Disease

**DOI:** 10.3390/cells9091966

**Published:** 2020-08-26

**Authors:** Helena Xicoy, Jos F. Brouwers, Bé Wieringa, Gerard J. M. Martens

**Affiliations:** 1 Department Cell Biology, Radboud Institute for Molecular Life Sciences, Radboud University Medical Centre, Geert Grooteplein Zuid 26-28, 6525 GA Nijmegen, The Netherlands; Be.Wieringa@radboudumc.nl; 2 Department Molecular Animal Physiology, Donders Institute for Brain, Cognition and Behaviour, Donders Centre for Neuroscience, Faculty of Science, Geert Grooteplein Zuid 26-28, 6525 GA Nijmegen, The Netherlands; g.martens@ncmls.ru.nl; 3 Department Molecular Cancer Research, Center for Molecular Medicine, University Medical Center Utrecht, Heidelberglaan 100, 3584 CX Utrecht, The Netherlands; j.f.h.brouwers-20@umcutrecht.nl

**Keywords:** Parkinson’s disease, RNA sequencing, transcriptomics, lipidomics, substantia nigra, putamen, postmortem human brain

## Abstract

Parkinson’s disease (PD) is characterized by the loss of dopaminergic neurons from the substantia nigra (SN) that project to the dorsal striatum (caudate-putamen). To better understand the molecular mechanisms underlying PD, we performed combined lipid profiling and RNA sequencing of SN and putamen samples from PD patients and age-matched controls. SN lipid analysis pointed to a neuroinflammatory component and included elevated levels of the endosomal lipid Bis (Monoacylglycero)Phosphate 42:8, while two of the three depleted putamen lipids were saturated sphingomyelin species. Remarkably, we observed gender-related differences in the SN and putamen lipid profiles. Transcriptome analysis revealed that the top-enriched pathways among the 354 differentially expressed genes (DEGs) in the SN were “protein folding” and “neurotransmitter transport”, and among the 261 DEGs from putamen “synapse organization”. Furthermore, we identified pathways, e.g., “glutamate signaling”, and genes, encoding, e.g., an angiotensin receptor subtype or a proprotein convertase, that have not been previously linked to PD. The identification of 33 genes that were common among the SN and putamen DEGs, which included the α-synuclein paralog β-synuclein, may contribute to the understanding of general PD mechanisms. Thus, our proof-of-concept data highlights new genes, pathways and lipids that have not been explored before in the context of PD.

## 1. Introduction

Parkinson’s disease (PD) is the second most common neurodegenerative disease, after Alzheimer’s disease. PD is rare in individuals less than 50 years old, but its prevalence increases with age, reaching up to 4% in older-age groups [1]. Clinically, PD presents with motor symptoms, e.g., rigidity, bradykinesia, tremor and postural instability [2], and non-motor symptoms, e.g., depression, hyposmia, constipation and sleep disorders [3]. The neuropathology of PD is characterized by the formation of Lewy bodies (abnormal intracellular aggregates containing metals, lipids and proteins like α-synuclein), a progressive loss of dopaminergic neurons that project from the substantia nigra (SN) to the dorsal part of the striatum (which consists of the caudate nucleus and putamen), and microgliosis [4].

Current knowledge of the molecular mechanisms underlying these pathological hallmarks has been derived from studies on 27 monogenic familial forms of PD [5], which represent only 5–10% of the cases, and from toxin-induced PD cellular and animal models (e.g., use of 1-methyl-4-phenyl-1,2,3,6-tetrahydropyridine, rotenone or 6-hydroxydopamine) [6,7,8,9]. The most salient mechanisms described up to now include mitochondrial dysfunction and oxidative stress, unfolded protein response, protein aggregation, lysosomal dysfunction and neuroinflammation [10]. Unfortunately, the existing data has not resulted in the development of disease-modifying treatments for PD.

Recent studies have pointed towards a key role for lipids in PD [11,12,13]. Based on their chemical and biochemical properties lipids can be classified into eight different classes, namely fatty acyls, glycerolipids, glycerophospholipids, sphingolipids, sterols, prenols, saccharolipids and polyketides [14]. Lipids are mainly known for their role in energy storage [15], but they are also involved in cellular signaling and transport [16,17]. Furthermore, lipids are the main constituents of cellular membranes [18], and form part of membrane rafts and anchors [19,20]. As such, an abnormal lipid composition has been linked to the molecular mechanisms underlying PD, i.e., protein aggregation [21], mitophagy [22] and immune processes [23].

Unbiased post mortem lipid profiling of brain tissue samples from PD patients and control individuals [24] could be a rich source of hypothesis-generating findings and key to further understanding of PD etiology. Of note, the lipidome is less susceptible to postmortem changes than the water-soluble metabolome and therefore the lipid composition of brain sections may offer a reliable representation of PD-related alterations in lipid homeostasis in vivo. Furthermore, genome-wide mRNA expression profiling, also known as transcriptomics, is one of the most-used -omics techniques and may be employed to shed light on how specific lipid changes are coupled to transcriptional regulation. Studies analyzing the PD brain transcriptome have mostly involved the use of microarrays [25]. Microarrays are more cost-effective than RNA sequencing (RNA-seq) [26], but the latter methodology is superior at detecting splicing events, and novel and/or low-abundance transcripts [27].

Here we perform lipid as well as RNA-seq analyses to determine the lipid and transcriptome profiles, respectively, of SN and putamen samples from the same PD patients and controls. Additionally, we compare our genome-wide mRNA expression findings to previous PD/control SN and putamen transcriptomic results to draw more robust conclusions about differential mRNA expression profiles in these PD brain regions.

## 2. Materials and Methods

### 2.1. Human Samples

Snap-frozen post-mortem human brain samples were obtained from the Netherlands Brain Bank (NBB; Project 870; Table 1) and all material and data collected by the NBB were obtained on the basis of written informed consent. The average age for control and PD individuals was 78.3 and 77.9 years old, respectively. The average postmortem delay (time interval from death to sample-freezing) for control and PD individuals was 6.49 and 5.53 h, respectively. The cerebrospinal fluid pH for control and PD individuals was 6.54 and 6.43, respectively. None of the three variables were significantly different between the groups (i.e., age: PD and controls (*p*-value > 0.999), male and female controls (*p*-value = 0.224), male and female PD (*p*-value = 0.386), males PD and controls (*p*-value = 0.892) and female PD and controls (*p*-value = 0.800); postmortem delay: PD and controls (*p*-value = 0.184), male and female controls (*p*-value = 0.191), male and female PD (*p*-value = 0.829), males PD and controls (*p*-value = 0.474) and female PD and controls (*p*-value = 0.200) and pH: PD and controls (*p*-value = 0.134), male and female controls (*p*-value = NA), male and female PD (*p*-value = 0.476), males PD and controls (*p*-value = 0.474) and female PD and controls (*p*-value = NA)). The cases reported here have not been genetically analyzed for familial mutations (5–10% of Parkinson cases are thought to be familial; [28]). All procedures were in accordance with the consensus criteria established by the Netherlands Brain Bank. Tissue blocks were cold grinded in liquid nitrogen to obtain a homogenous sample for both RNA and lipid extraction.

### 2.2. Lipid Profiling

Lipid profiling concerned a validated method for reproducible and high-throughput lipid analysis as described in [29,30,31]. Briefly, pellets of grinded tissue samples were mixed with UPLC-grade chloroform: methanol 1:1 (*v*/*v*) and, after 20 min, samples were centrifuged at 2000× *g* and the supernatant was used directly for LC–MS analysis. To this end, 10 µL was injected on a hydrophilic interaction liquid chromatography (HILIC) column (2.6 µm HILIC 100 Å, 50 mm × 4.6 mm, Phenomenex, Torrance, CA, USA), and eluted with a gradient from ACN/Acetone (9:1, *v*/*v*) to ACN/H2O (7:3, *v*/*v*) with 10 mM ammonium formate, and both with 0.1% formic acid. Flow rate was 1 mL/min. The column outlet of the LC was either connected to a heated electrospray ionization source of an LTQ-XL mass spectrometer or a Fusion mass spectrometer (both from ThermoFisher Scientific, Waltham, MA, USA). Full scan spectra were collected from *m*/*z* 450–950 at a scan speed of 3 scans/s in both the positive and negative ionization mode (LTQ-XL). On the Fusion mass spectrometer full spectra were collected in the negative ionization mode from *m*/*z* 400 to 1600 at a resolution of 120,000. Parallel data dependent MS2 was done in the linear ion trap at 30% HCD collision energy. Retention times of lipid classes were verified by comparison to authentic standards and response factors of lipid classes were calculated. Care was taken to ensure that lipid concentrations did not exceed the linear response range of the instrument.

### 2.3. RNA-Sequencing Analysis

RNA was extracted using the RNeasy Lipid Tissue Mini Kit (Qiagen, Cat No./ID: 74804, Venlo, The Netherlands). Total RNA samples were spectrophotometrically analyzed, and their 260/280 ration was typically above 1.9. RNA-seq directionality library preparation and sequencing with Poly(A) selection was performed by HudsonAlpha Genomic Services Laboratory (Huntsville, AL, USA). Samples underwent sequencing on the HiSeq v4 (PE, 50 bp, 25 M reads), per standard protocols. Sixteen samples were run over two lanes (3.125 M reads/sample). Data is deposited under the accession number GSE136666.

Quality control from raw data was performed with the FASTQC tool (https://www.bioinformatics.babraham.ac.uk/projects/fastqc/, Babraham Institute, Cambridge, UK). Overall, data had enough quality to follow with transcriptome alignment. Reads mapping and quantification was performed with the Salmon pipeline [32], which consists of a lightweight-mapping model, an online phase that estimates initial expression levels and model parameters, and an offline phase that refines expression estimates. Next, samples with less than 10 reads across samples were filtered out. Quality controls, including sample distances and principal component analysis, performed after variance stabilizing transformation, concluded that all samples could be included for the differential expression analysis. Differential expression analysis was performed with the DESeq2 v1.22.2 Bioconductor’s package [33,34]. Finally, clusterProfiler [35] was used to analyze and visualize functional profiles.

### 2.4. Compilation of Transcriptomic Studies

The terms used to browse the GEO DataSets database included “Parkinson’s disease” and “Substantia Nigra” or “Putamen”. GEO DataSets was applied to screen the studies compiled by [25]. Thirteen studies on human postmortem SN and/or putamen samples were found (Table 2) and they were used for the validation of our data, except for the study that distinguished between medial and lateral SN (GSE8397) was excluded.

### 2.5. Gene Set Enrichment Analysis

The bioconductor limma parametric multivariate rotation gene set test (ROAST) [37] was applied to evaluate gene sets associated with the metabolism and/or cellular signaling pathways of the lipid classes deregulated in the SN and putamen. The terms “phosphatidylcholine”, “phosphatidylethanolamine”, “phosphatidylserine”, “phosphatidylinositol” and “sphingomyelin” were searched at AmiGO [38] and Harmonizome [39]. From the results obtained, the gene sets with (1) only the lipid class name, (2) metabolism, biosynthesis and catabolism or (3) binding information, and containing at least 3 genes, were included in the analysis. Additionally, the specific lipid species were searched in the Human Metabolome Database [40] and retrieved the related proteins, which were also included in the analysis. More precise statistical results were obtained by running the analysis with 10,000 random rotations, and significance values were corrected for multiple testing using the Benjamini–Hochberg false discovery rate method.

### 2.6. Experimental Design and Statistical Analyses

In this exploratory proof-of-concept study, twenty post-mortem human SN samples (10 controls and 10 PD patients) and 18 post-mortem striatum samples (9 controls and 9 PD patients), from the same individuals, were analyzed. Age, postmortem interval and cerebrospinal fluid pH differences between groups were assessed by the non-parametric Mann–Whitney U test. Lipid data analysis was performed by combining a univariate analysis and an ensemble learning method, together with a Receiver Operating Characteristic (ROC) analysis to select optimal findings. The univariate analysis included multiple comparisons of all lipids and a selection of those with a *p*-value < 0.05. Random forests, an ensemble learning method, were used to identify lipids relevant for disease/gender classification. Similar to a previous study [41], the classification at each node in a tree was done with 100 randomly selected metabolites and the forest consisted of 5000 trees. The process was repeated 50 times, and the model with the highest area under the curve (AUC) was kept. Lipids with a mean decreased accuracy >2 were considered relevant. A ROC analysis was performed on all the lipids considered relevant by either method, and an AUC > 0.8 and *p*-value < 0.05 was established as the threshold for important lipids to distinguish between conditions. Lipids that fulfilled at least two out of the three criteria were considered as associated with the disease/gender.

For RNA-seq analysis, pools of two and three RNA samples of the same gender and condition were made for the SN and putamen samples, respectively. Differential expression analysis was performed with the DESeq2 v1.22.2 Bioconductor’s package [33,34]. Since this analysis represents an exploratory study, we chose not to use multiple testing adjustments, as supported by several authors [42], but apply a threshold for effect size. Hence, genes with a *p*-value < 0.01 and > 0.5849-log2FC were considered as significant.

Regarding the reanalysis of published transcriptomics data, all included datasets were analyzed using GEO2R [43] to obtain a homogeneous analysis. Genes with *p*-value < 0.01 and log2FC < −0.5849 or log2FC > 0.5849 were considered differentially expressed genes (DEGs). To convert the obtained identifiers to gene symbols when these were not available from the GEO2R analysis, g:Profiler (version e95_eg42_p13_f6e58b9, University of Tartu, Tartu, Estonia) was used [44]. Two studies did not report any DEGs and one study had only upregulated DEGs, and the three studied were therefore excluded.

## 3. Results

### 3.1. Lipid Profiling

**Figure 1:**
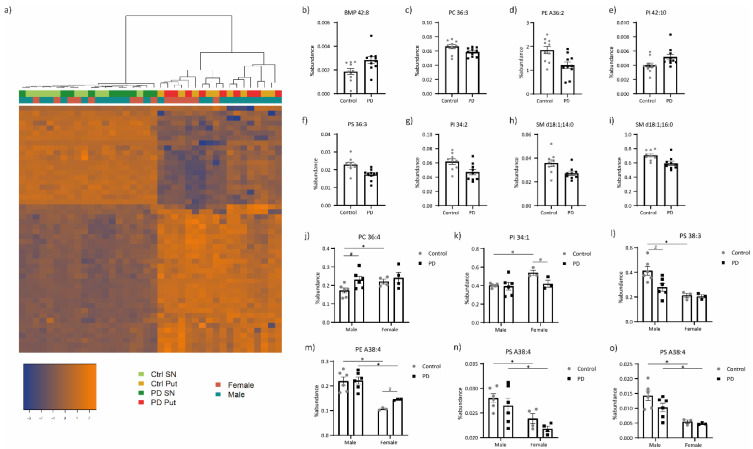
Lipid profiling of the SN and putamen from PD patients compared to controls. (**a**) Hierarchical clustering of the samples based on the different abundances of lipid species. Percentage of abundance of lipid species that differ between PD patients and controls, according to at least two of the three selection criteria, in (**b**–**f**) SN and (**g**–**i**) putamen, and between male and female controls, and also in (**j**) SN and (**k**) putamen of male PD patients compared to controls, or in (**l**) putamen of female PD patients compared to controls. Percentage of abundance of a lipid that (**m**) has different abundances in both males and female controls, and male and female PD patients, together with a difference in female PD patients compared to controls, and that is lower in females than in males, both in controls and PD patients, in (**n**) SN and (**o**) putamen. BMP, Bis(Monoacylglycero)Phosphate; PC, phosphatidylcholine; PE, phosphatidylethanolamine; PI, phosphatidylinositol; PS, phosphatidylserine and SM, sphingomyelin, where d18:1 indicates the common sphingosine base c.f. standard nomenclature [45]. Sample values are plotted individually. ‘A indicates that the sn-1 position is linked via an ether linkage. Numbers (X:Y) represent the number of carbons (X) and double bonds (Y). # Differences between control and PD samples, within one gender; * differences between male and female samples, within one condition.

The lipid compositions of the SN and putamen samples from PD patients and controls were analyzed by LC–MS. We identified 269 phospholipids of the following classes: Bis (Monoacylglycero)Phosphate (BMP), phosphatidylcholine (PC), phosphatidylethanolamine (PE), phosphatidylglycerol (PG), phosphatidylinositol (PI) and phosphatidylserine (PS), and the sphingolipid sphingomyelin (SM; Spreadsheet S1). Hierarchical clustering showed a clear separation of the lipid patterns between the samples from the two brain regions, but not a clear division between the samples based on pathology or gender (Figure 1a). In the SN, we found five lipid species that have different abundances between PD patients and controls, namely BMP 42:8, PC 36:3, PE A36:2, PI 42:10 and PS 36:3 (Figure 1b–f), while three differentially expressed lipid species were identified in the putamen, i.e., PI 34:2, SM d18:1;14:0 and SM d18:1;16:0 (Figure 1g–i).

Since sex hormones have been found to modulate brain lipid levels [46], we performed the analyses separately for males and females (Spreadsheet S1). Interestingly, a number of lipid species displayed different abundances in female and male controls, including PC 36:4 in the SN (Figure 1j), and PI 34:1 and PS 38:3 in the putamen (Figure 1k,l), while these differences were not present in PD patients. Additionally, some lipid species had distinct levels in female and male controls and PD, e.g., PE A38:4 in the putamen (Figure 1m), whereas PS A38:4 levels were lower in females than in males, regardless of the condition and in both brain regions (Figure 1n,o).

### 3.2. Transcriptomic Profiling

**Figure 2:**
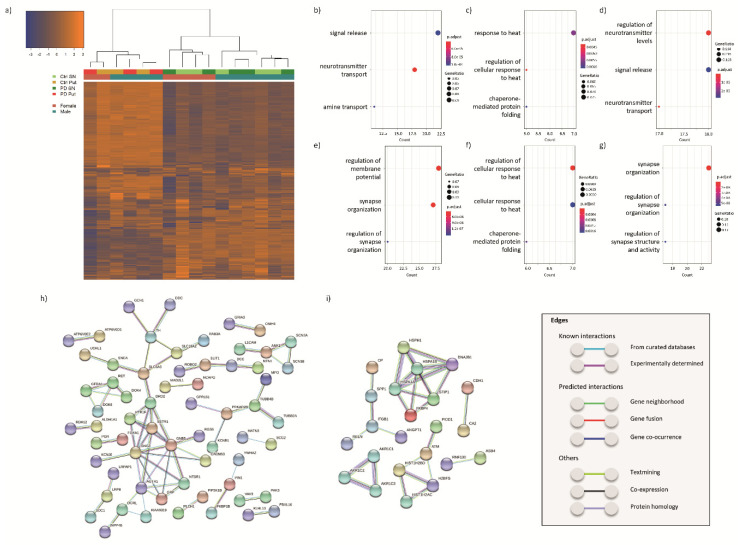
Transcriptomic analysis of the SN and putamen from PD patients compared to controls. (**a**) Hierarchical clustering of the samples based on the transcriptomic results; biological pathways enriched in (**b**) all, (**c**) upregulated and (**d**) downregulated DEGs found in our RNA-seq analysis of SN and in (**e**) all, (**f**) upregulated and (**g**) downregulated DEGs from the overlap between previous microarray mRNA expression profiling studies on SN from PD patients and controls (GSE7621, GSE42966, GSE49036, GSE20164, GSE20163, GSE20333 and GSE54282), including also the present study; interaction networks of proteins encoded by genes (**h**) downregulated or (**i**) upregulated in at least two of the eight studies included in the overlap analysis. Protein–protein networks were obtained with STRING [31]. Only the highest confidence associations were included. Non-connecting nodes were removed.

We performed RNA-seq analysis of SN and putamen samples from PD patients and age-matched controls. Hierarchical clustering showed a clear separation of the transcriptomic profiles between the samples from the two brain regions, as well as between the male and female samples (Figure 2a).

#### 3.2.1. DEGs in the SN of PD Patients and Controls

We then performed a restrictive analysis of the RNA-seq data (adjusted *p*-value < 0.05 and >0.5849-log2FC) and identified 39 upregulated and 41 downregulated transcripts (Spreadsheet S2). Among the DEGs encoding these transcripts, 7 represented unknown genes (no gene symbol associated), 2 were uncharacterized transcripts (LOC) and 3 were genes for long intergenic non-protein coding RNAs (lincRNAs). The top 5 upregulated DEGs were *OR7C1* (log2FC = 2.05), *MTRNR2L8* (log2FC = 1.94), processed pseudogene *MTCYBP18* (ENSG00000244921, log2FC = 1.94), *PCDH20* (log2FC = 1.61) and *KLF5* (log2FC = 1.50), while the top 5 downregulated DEGs were *TPH2* (log2FC = −1.98), *LOC101929445* (log2FC = −1.96), *ADRA1D* (log2FC = −1.63), *IL1B* (log2FC = −1.62) and *DOK7* (log2FC = −1.58).

Next, we performed a less-restrictive RNA-seq data analysis (*p*-value < 0.01 and > 0.5849-log2FC) to determine which biological pathways were enriched in the dataset. This analysis yielded 354 DEGs in the SN of PD patients compared to controls, of which 152 were upregulated and 202 downregulated (Spreadsheet S2). To understand the biological significance of this outcome, we analyzed on the basis of gene ontology (GO) terms which biological pathways were enriched in the 354 DEGs as well as in the up- and down-regulated DEGs separately (Spreadsheet S2). The top-three pathways in the all-DEGs analysis were “signal release” (GO:0023061), “neurotransmitter transport” (GO:0006836) and “amine transport” (GO:0015837; Figure 2b). The pathways “response to heat” (including GO:0009408 and GO:1900034) and “chaperone-mediated protein folding” (GO:0061077) represented the top significantly enriched pathways in the upregulated genes (Figure 2c), while the biological pathways related to “regulation of neurotransmitter levels” (GO:0001505), “signal release” (GO:0023061) and “neurotransmitter transport” (GO:0006836) were the top significantly enriched ones among the downregulated DEGs (Figure 2d).

Subsequently, we validated our results by comparison with the results of the only other RNA-seq study on PD and control SN [47]. We found that 9.8% of the DEGs identified in our study were also listed as differentially expressed in [47]. However, while 47% of the overlapping genes were differentially expressed in the same direction, the remaining 53% were differentially expressed in the opposite direction, questioning the validity of the overlap. We then aimed to validate our results and obtain more robust outcomes by comparing the publicly available transcriptomic studies performed with microarrays, homogeneously analyzed with GEO2R, and our data. Interestingly, our data partially overlaps with the results of these previous studies (Table 3). We found that 311 DEGs (112 upregulated and 199 downregulated) were present in the same direction in at least two datasets (overlapping genes; Spreadsheet S3). The top biological pathways enriched in the 311 DEGs, and also in the downregulated DEGs, were associated with “synapse organization” (e.g., GO:0050808), while the top pathways enriched in the upregulated DEGs were “regulation of cellular response to heat” (GO:1900034) and “chaperone-mediated protein folding” (GO:0061077; Figure 2e–g).

Next, we analyzed the interactions between the proteins encoded by the 311 DEGs found in at least two studies (overlap). One of the largest clusters of proteins encoded by the downregulated DEGs (Figure 2h), which includes some of the DEGs common in four out of eight studies, such as *TH*, *DDC*, *DRD2*, *KCNJ6* (also known as GIRK2), *SLC18A2* (VMAT2) and *SLC6A3* (DAT), was related to dopamine synthesis and transport, providing confidence in the effectiveness of our approach. This pertinent cluster was also associated with other proteins linked to the G-protein-coupled receptor signaling (e.g., *GNG3*). Additional protein clusters linked to downregulated DEGs were associated with GDNF-RET signaling (e.g., *RET*), and neural development (e.g., *SLIT1*). The protein–protein interaction networks of upregulated DEGs (Figure 2i) consisted of six main protein clusters associated with multiple cellular processes, including protein folding (e.g., *HSPA1A*), the aldo-keto reductase family (e.g., *AKR1C1*), cell migration and extracellular matrix (e.g., *ITGB1*), DNA (damage) (e.g., *ATM*) and ubiquitination (e.g., *RNF130*).

#### 3.2.2. DEGs in the Putamen of PD Patients and Controls

We identified 15 DEGs (5 upregulated and 10 downregulated genes) in the putamen of PD patients compared to controls when using restrictive parameters for RNA-seq data analysis (adjusted *p*-value < 0.05 and > 0.5849-log2FC). Two of these DEGs encoded unknown transcripts (no gene symbol associated) and 1 lincRNAs RNA, respectively. The five upregulated DEGs produced to two unknown transcripts (ENSG00000244921, log2FC = 2.22, and ENSG00000257767, log2FC = 1.41) and transcripts for *MYOT* (log2FC = 0.97), *ZNF646* (log2FC = 0.74) and *HIP1BP3* (log2FC = 0.60), while the top five downregulated DEGs were for *SLC30A3* (log2FC = −2.26), *DUSP2* (log2FC = −2.10), *PPL* (log2FC = −1.68), *GRM2* (log2FC = −1.67) and *GNG2* (log2FC = −1.58).

Next, we identified 261 DEGs in the putamen of PD patients compared to controls with the less-restrictive analysis (*p*-value < 0.01 and > 0.5849-log2FC), of which 68 were upregulated and 193 downregulated (Spreadsheet S4). The top three pathways enriched in these putamen DEGs were “synapse organization” (GO:0050808), “cartilage development” (GO:0051216) and “prostaglandin transport” (GO:0015732). There were no biological pathways enriched in the upregulated DEGs, while the downregulated ones were involved in “synapse organization” (GO:0050808), “cartilage development” (GO:0051216) and “synapse assembly” (GO:0007416; Spreadsheet S4).

There are less transcriptomic studies on putamen than SN from PD patients and controls, and most of them have resulted in the identification of only a very low number of DEGs. Moreover, essentially no overlap between the DEGs has resulted from these studies (Table 4). We therefore performed no further analysis on these datasets.

#### 3.2.3. DEGs Common between SN and Putamen

Finally, we analyzed the overlap between SN and putamen mRNA expression profiles to find possible global mechanisms underlying PD. As mentioned above, since there was no robust overlap between previous transcriptomic studies on the putamen, we could only use our own data and found 33 DEGs common between the SN and putamen profiles (Table 5).

### 3.3. Integration of the Lipid and Transcriptomic Profiling Data

Since the lipid and transcriptomic profiling data were obtained from the same samples, we analyzed if gene sets linked to the deregulated lipids (i.e., BMP 42:8, PC 36:3, PE A36:2, PI 42:10 and PS 36:3 in the SN, and PI 34:2, SM d18:1;14:0 and SM d18:1;16:0 in the putamen) were differentially expressed. Accordingly, we performed the targeted gene set enrichment ROAST test, a hypothesis-driven analysis (Table 6). A weak association (*p*-value < 0.05 and FDR-adjusted *p*-value < 0.25) between the upregulated SN transcripts and the dataset “phosphatidylcholines” (GeneRIF), and between the deregulated SN transcripts and the datasets “phosphatidylcholine biosynthetic process” (GO:0006656) and “phosphatidylcholine catabolic process” (GO:0034638) were found. No (weak) association between the deregulated transcripts from the SN and phosphatidylinositol-related gene sets was observed. Furthermore, a weak association between the deregulated SN transcripts and the datasets “phosphatidylethanolamine biosynthetic process” (GO:0006646) and “phosphatidylethanolamine biosynthesis” (HumanCyc Pathways) were found. Additionally, a weak association between the upregulated SN transcripts and “phosphatidylserine binding” (GO:0001786), and between the deregulated SN transcripts and the dataset “phosphatidylserine catabolic process” (GO:0006660) were found. A weak association between downregulated transcripts in the putamen and the datasets “phosphatidylinositol binding” (GO:0035091) and “Sphingomyelin Metabolism/Ceramide salvage” (HumanCyc Pathways) was also found. Unfortunately, retrieval of gene sets associated with the poorly characterized lipid BMP 42:8 was not possible.

## 4. Discussion

The data presented here represent the first lipid and transcriptome analyses performed on PD and control SN as well as putamen samples from the same individuals. We conducted a transcriptome analysis by RNA-seq rather than microarrays in order to identify not only annotated transcripts, but also currently unannotated sequences for future re-evaluation (50/354 SN DEGs and 29/261 putamen DEGs represent not-annotated transcripts) [26]. Additionally, we analyzed our data together with publicly available transcriptome datasets to draw more robust conclusions about the relevance of gene functions and pathways for PD.

Lipidomics likely represents the most informative -omics technology complementary to RNA-seq. In this context, it is indeed important to realize that lipids are structural and bioactive molecules essential for multiple brain processes such as myelination, synaptic signal transduction and acting as second-messenger precursors and, relative to water-soluble metabolites, generally have a much slower turnover and are thus less prone to post-mortem degradation. Previously, neuropathological lipidomic studies have been conducted on primary visual cortex [48], primary motor cortex [49], hippocampus [50] and cerebellum [51] from PD and/or Lewy body disease individuals. Our exploratory lipid profiling involved the analysis of seven classes of lipids and showed several lipid species modulated in the SN and putamen of PD patients compared to age-matched controls. Two other studies have been performed on the lipid profile of the SN [52,53] and one on the putamen [51] of PD patients, but they have reported only differences in lipid classes rather than lipid species and focused on sphingolipid and gangliosides, respectively, hindering a comparative analysis. The differences we found in the putamen include PI 34:2 and two saturated SM species, namely SM d18:1;14:0 and SM d18:1;16:0. Unfortunately, the limited current knowledge of putamen lipids does not allow a functional interpretation of the putamen lipid changes we detected. Lipids BMP 42:8 and PI 42:10, both with increased abundance in the SN of PD patients compared to controls, are thought to have arachidonic acid (20:4) as one of the two side chains, while the three decreased lipid species, PC 36:3, PE A36:2 and PS 36:3, are likely to have linoleic acid (18:1) as one of their two side chains. Although such specific changes in lipid composition are difficult to directly link to distinct enzymatic activities in specific metabolic conversion steps, it is tempting to speculate on their wider biological implications. The presence of the five differentially expressed SN lipid species could point towards a neuroinflammatory component in disease etiology, since increased arachidonic acid has been demonstrated in acute neuroinflammation [54], while linoleic acid is one of the sources of arachidonic acid [55]. A neuroinflammatory involvement is also in line with the findings of our transcriptomic analysis revealing DEGs associated with the transport of prostaglandins, which regulate neuroinflammatory pathways. Lipid BMP 42:8 belongs to a lipid class highly enriched in lysosomal membranes [56] and is key for the proper functioning of lysosomal hydrolases [57]. Interestingly, BMP accumulation also occurs in macrophages from patients with Gaucher disease [58], a rare genetic disorder characterized by deposition of the lipid glucocerebroside in macrophages and that highly increases the life-time risk to develop PD [59]. Nevertheless, we realize that it is difficult to draw functional conclusions concerning individual lipids since the number of samples employed in this proof-of-concept study is relatively small and only the roles of lipid classes have been characterized.

Even though our sample size was small, it is important to note that we found gender-related differences in the abundance of lipids both in PD patients and controls. This observation is consistent with the gender-related differences that have been found in the SN lipid profiles of PD patients [53] and the “female pregnancy” pathway detected in our transcriptional analysis. Current data indicates that gender differences exist not only in PD incidence and prevalence [60], but also in its clinical manifestation [61,62,63], with estrogens as a possible mediator for these differences [64], although the underlying molecular mechanisms are unknown. Since estrogens are steroid hormones and thus lipids modulating lipid metabolism in the brain [65], and gender dimorphism has been observed in the fatty acid profiles of control mouse brains [66] as well as in our data, brain lipidome modulation by sex hormones may be one of the underlying protective mechanisms in PD. Clearly, the possible link between sex hormones, brain lipids and PD deserves further attention.

In our exploratory transcriptomic study, the top upregulated DEG in the SN was *ORC7C1*, which encodes an odorant receptor. Mesencephalic dopaminergic neurons express olfactory receptors and they respond to odorant-like molecules in mice [67]. Several odorant receptors have been found to be downregulated in the frontal cortex of PD patients [68], but the difference in the directionality of expression (up- or down-regulation) could be brain region related. The fact that the top upregulated DEG as well as the 7th upregulated DEG (i.e., *OR7A5*) represent odorant receptors highlights the importance of a poorly studied group of receptors that could modulate the cell behavior and fate via binding of small molecules and, thus, could play a crucial role in PD. The relationship between the upregulated *MTRNR2L8* and the mitochondrial *MT-RNR2* is at present unclear [69] and its role in PD has not been analyzed yet. The protocadherin encoded by *PCDH20*, which is thought to be involved in the establishment and function of specific cell–cell connections in the brain, has been found to be also upregulated in a meta-analysis of transcriptomic studies from SN of PD patients [70]. Nevertheless, the role of this protein has thus far not been studied in the context of PD. The transcription factor KLF5 binds to GC box promoter elements and activates their transcription, mediating multiple cellular processes, such as proliferation, migration and differentiation, among others [71], but its significance for PD is currently unknown.

The top-downregulated DEG in the SN is *TPH2*, which encodes the rate-limiting enzyme in the synthesis of serotonin. Interestingly, serotoninergic neurons are progressively lost in PD, which is implicated both in motor and non-motor manifestations of PD [72]. Moreover, polymorphisms in *TPH2* are associated with addictive behaviors [73] and depression [74] in PD patients. Additionally, Tph2 KO mice, which have serotonin deficiency, present with systemic oxidative stress and lipidomic abnormalities [75], and swallowing dysfunction [76]. The alpha-D1 adrenergic receptor encoded by *ADRA1D* is also downregulated in the hippocampus of Alzheimer’s disease and dementia with Lewy bodies patients [77], and noradrenergic impairment is present in PD patients as well [78]. Both findings highlight the importance of neurotransmitters other than dopamine in the pathology of PD. It is therefore imperative to do further studies into their role to obtain a better understanding of the complexity of disease. The observed downregulated expression of *IL1B* is in contrast with earlier findings showing increased *IL1B* expression in striatum [79] and cerebrospinal fluid [80] of PD patients. However, the complex effects of IL1B seem to depend on the time, place and level of *IL1B* expression, e.g., infusion of IL1B in the striatum of rats 5 days before 6-OHDA injection had a protective effect [81]. Finally, *DOK7* encodes a protein involved in neuromuscular synaptogenesis, but its role in PD pathogenesis remains to be established.

PD is characterized by the loss of dopaminergic neurons from the SN [4], which reaches a reduction of at least 70% by the time the disease is diagnosed. Thus, our top finding of decreased expression of genes associated with neurotransmitter levels, and dopamine synthesis and transport in postmortem PD SN samples is likely linked to the decreased number of dopaminergic neurons and fibers in the SN and putamen, respectively. One of these was the PD-associated *RET* gene [82], encoding a GDNF receptor required for the preventive and compensatory mechanisms linked to dopaminergic system degeneration that is triggered by the neurotrophic factor [83,84]. Remarkably, RET and some of its interaction partners (GFRA1, DOK4 and DOK6) are downregulated in multiple SN transcriptomic studies, highlighting the importance of the process of dopaminergic neurodegeneration in PD brains. The two most-enriched upregulated pathways were “cellular response to heat” and “protein folding”, which have an overlap of 80% among their DEGs. Since misfolded proteins are a hallmark of PD [85], upregulation of genes associated with these pathways, such as the molecular chaperone HSPA1A, may represent a compensatory cellular mechanism to handle the accumulation of misfolded proteins. Furthermore, we confirmed other previously reported PD mechanisms, such as a dysfunction of protein folding. Interestingly, other SN DEGs comprise novel PD-related pathways that may provide a better understanding of the disease pathology, including “positive regulation of acute inflammatory response” (GO:0002675), “female pregnancy” (GO:0007565) and “G-protein-coupled glutamate receptor signaling pathway” (GO:0007216).

The overlap of our RNA-seq data with results from earlier microarray studies on postmortem PD and control SN confirms the importance of the degeneration of dopaminergic neurons in this brain region, since 10 out of the 12 DEGs that were found in at least half of the studies are associated with dopaminergic neurons or PD (*ALDH1A1*, *DDC*, *DLK1*, *DRD2*, *GAP43*, *KCNJ6*, *SLC18A2*, *SLC6A3*, *SV2C* and *TH*). The other two genes are *AGTR1*, the angiotensin II receptor subtype AT1, which has been shown to be downregulated in 5 out of the 8 SN studies, and the neuroendocrine proprotein convertase 1, *PCSK1*, which was downregulated in 4 out of the 8 SN studies. *AGTR1* and *PCSK1* do not have a known role in dopaminergic neurons or PD and are therefore interesting candidates for future functional studies. The reason for the restricted overlap between the results of our study and those of the only other PD/control SN RNS-seq analysis is at present unclear.

The top upregulated protein-encoding transcript in the putamen is MYOT, which codes for a component of a complex of actin cross-linking proteins. Transcript levels of MYOT have been found to be upregulated and downregulated in the SN and blood of PD patients, respectively [86]. Furthermore, PD patients present a higher prevalence of MYOT autoantibodies than controls [87]. Thus, MYOT may play distinct roles in the pathology of PD, but the nature of its involvement has not been elucidated. Neither have the function of the transcription factor ZNF646 or its link to PD been widely studied. The upregulated gene *HP1BP3* mediates chromatin condensation and modulates cognitive aging [88], but not in known association with PD.

The top downregulated transcript in the putamen is SLC30A3, which encodes a protein involved in the accumulation of zinc in synaptic vesicles, rather than in the cytosol. Decreased levels of the protein have been observed in the (pre)frontal cortex of DLB and PDD patients [89,90] and is thus associated with dementia. Moreover, PD patients present changes in the cellular distribution of zinc in the SN [91] and treatment of midbrain primary cultures with MPP+ leads to zinc ion accumulation [92]. These findings highlight the relevance of bivalent metal ions in the pathology of PD and encourage further studies in this direction. The second most downregulated transcript was DUP2, a phosphatase involved in the negative regulation of ERK1 and ERK2. Members of the ERK family of proteins are known to control cellular proliferation and differentiation, but association of this function with PD has not yet been found. Similarly, *GNG2* encodes a gamma subunit of heterotrimeric G proteins, which are involved in cellular responses to external signals, but the significance of this function for PD still needs to be clarified. *PPL* codes for a component of desmosomes and is thus involved in filament binding. Although *PPL* has not been associated with PD, genetic variants of another Plakin family member, *MACF1*, lead to decreased expression and confer risk for PD [93]. Finally, *GRM2* encodes a metabotropic glutamate receptor and, interestingly, glutamate-induced excitotoxicity has been suggested to result in the loss of dopaminergic cells in PD [94]. Moreover, activation of both metabotropic glutamate receptors 2 and 3, which have inhibitory functions, gives rise to striatal protection in PD rodent models [95]. Hence, this finding can be considered further support for the involvement of multiple neurotransmitter systems in PD.

Analysis of data from various transcriptomic studies on putamen showed no overlapping genes. While SN is considered to be one of the primary sites of degeneration in PD, the neurodegeneration in the dorsal striatum may be mainly a consequence of the depletion of dopaminergic innervation [96]. Thus, the lack of overlap among putamen DEGs could point to more heterogeneous pathology in the putamen than in the SN. We found an enrichment of downregulated putamen genes that play a role in the control of neurotransmitter levels and transport, which may well be associated with the loss of dopaminergic innervation [96]. Furthermore, we observed DEGs associated with prostaglandin transport, which may argue for a neuroinflammatory response in this brain region [97]. Indeed, neuroinflammatory events may play a role in nearly all known neurodegenerative diseases [98].

Interestingly, we found 33 genes that were differentially expressed in both the PD SN and putamen, hinting at mechanisms shared by the two brain regions. The use of pathway analysis software nor the findings from extensive literature searches led to the identification of a specific pathway within the set of 33 genes. Of note is the downregulation of β-synuclein, the paralog of α-synuclein, that inhibits the assembly [99], aggregation [100] and toxicity [101,102] of α-synuclein. Since α-synuclein aggregation is one of the neuropathological hallmarks of PD [103], β-synuclein upregulation may represent a potential target to ameliorate the disease. Hence, overexpression of β-synuclein in cellular and animal PD models would be central to better understand its role in the disease. Other potentially interesting examples of the set of common SN and putamen DEGs include *DDIT4*, which mediates apoptosis in cellular PD-models [104], and *P2RX7*, the antagonist of which is neuroprotective in cellular and animal models for PD [105]. Additionally, SNPs in the common genes encoding *EDN1* and *MYO5B* have been associated with multiple systems atrophy (which presents with parkinsonism [106]) and loss of the sense of smell (an early non-motor PD-symptom [107]), respectively. Therefore, functional studies on the genes that are differentially expressed in both brain regions, e.g., by manipulating their expression in cellular and animal PD models, may provide clues for further understanding of the pathobiological complexity of PD.

The parallel analysis of the lipid and transcriptomic profiles of the same samples allowed us to integrate the two datasets. In this connection, one has to realize that the characterization of lipid species is limited, while the proteins involved in the metabolism of lipid classes have been well studied. Yet, targeted gene set enrichment analysis revealed that gene sets associated with the lipid classes differentially expressed in the SN and putamen were deregulated in the respective brain region. Thus, the lipidome and the transcriptome appear to be tightly connected, and transcriptomic changes leading to differences in the lipid profile may be one of the molecular mechanisms underlying neurodegeneration in PD. Still, additional studies involving multiple -omics approaches are necessary to allow a better characterization of the role of lipids in PD. Such studies will be of particular interest since several familial PD genes, such as *LRRK2* [108], *PINK1* [109], *DJ1* [110], *ATP13A2* [111], *PLA2G6* [112] and *ATXN2* [113], are known to modulate various lipid metabolism pathways.

This study has several limitations. First, relative to control samples, the SN samples from PD patients are characterized by an extensive loss of dopaminergic neurons and gliosis, which hinders making a distinction between lipid/mRNA changes caused by the disease-generated differences in the composition of cellular populations or by the molecular mechanisms leading to neurodegeneration (i.e., the “consequence” or the “cause”). Second, our lipid analysis was focused on phospholipids and sphingolipids, and did not include other lipid classes that could be relevant for PD, such as sterols and glycolipids [24]. Third, we pooled samples for RNA-seq analysis, which hampered a gender-dependent analysis as performed with the lipid profiling, although the hierarchical clustering showed a clear separation by gender. Moreover, the pooling of samples might have masked minor differences in mRNA expression levels due to a small sample size and thus less statistical power.

## 5. Conclusions

We here reported the first combined lipid and transcriptomic analyses of SN and putamen samples from the same PD patient and control individual. This exploratory proof-of-concept study allowed us to unravel changes in the PD brain lipid profile, some of which were gender dependent and should be studied further to evaluate their relevance. Additionally, we confirmed molecular pathways associated with PD and identified several potentially interesting novel genes and pathways that may help to better understand PD pathology.

## Figures and Tables

**Table 1:** Clinical information on the post-mortem human brain samples.

Diagnosis	Age	Gender	PMD	pH CSF	Braak Stage	Cause of Death
Control	78	M	8:25	6.56	0	Cardiac arrhythmia
Control	69	F	8:30		0	Myocardial infarction
Control	69	F	6:15	6.59	0	Cardiogenic shock
Control	84	M	7:05	5.90	0	Exacerbation of COPD
Control	73	F	6:40		0	Respiratory failure
Control	84	M	5:35	6.98	0	Heart failure
Control	82	M	5:10	6.75	0	Pneumonia
Control	85	F	7:05		0	Renal insufficiency
Control	79	M	5.45	6.38	0	Euthanasia
Control	80	M	4:25	6.59	0	Euthanasia
PD	86	F	4:08	6.32	5	Cachexia and dehydration
PD	86	M	7:25	6.26	4	Cardiac arrest
PD	74	M	4:35	6.58	6	Respiratory insufficiency
PD	68	F	4:05		6	Euthanasia
PD	77	M	3:10	6.28	6	Aspiration pneumonia
PD	86	M	4:10	6.91	6	Euthanasia
PD	84	M	4:50	6.41	3	Cachexia
PD	76	M	9:15	6.33	6	Ileus
PD	77	F	6:05	3.20	5	Stroke
PD	65	F	7:35	6.55	6	Cachexia and dehydration

The table includes the diagnosis (control or Parkinson’s disease (PD)), age at death, gender (male (M) or female (F)), post-mortem delay (PMD), pH of the cerebrospinal fluid (CSF), Braak stage and cause of death.

**Table 2:** Summary of publicly available transcriptomic studies on human postmortem substantia nigra (SN) and/or putamen from PD patients and controls.

Accession Number	Tissue	Control	PD Patient	Platform	Inclusion
GSE7621	SN	9	16	[HG-U133_Plus_2] Affymetrix Human Genome U133 Plus 2.0 Array	Yes
GSE42966	SN	6	9	Agilent-014850 Whole Human Genome Microarray 4x44K G4112F	Yes
GSE43490	SN	5	8	Agilent-014850 Whole Human Genome Microarray 4x44K G4112F	No (only overexpressed genes)
GSE49036	SN	8	15	[HG-U133_Plus_2] Affymetrix Human Genome U133 Plus 2.0 Array	Yes
GSE20164	SN	5	6	[HG-U133A] Affymetrix Human Genome U133A Array	Yes
GSE20163	SN	9	8	[HG-U133A] Affymetrix Human Genome U133A Array	Yes
GSE20292	SN	18	11	[HG-U133A] Affymetrix Human Genome U133A Array	No (no DEGs)
GSE20333	SN	6	6	[HG-Focus] Affymetrix Human HG-Focus Target Array	Yes
GSE8397	SNm	7	9	[HG-U133A] Affymetrix Human Genome U133A Array; [HG-U133B] Affymetrix Human Genome U133B Array	No (two different SN tissues (medial and lateral))
	SNl	8	15		
GSE54282	SN	3	3	[HuGene-1_0-st] Affymetrix Human Gene 1.0 ST Array [HuGene10stv1_Hs_ENTREZG_15.0.0]	Yes
	Put	6	6		Yes
GSE77666	Put	12	12	NanoString nCounter gene expression system	Yes
GSE23290	Put	5	5	[HuEx-1_0-st] Affymetrix Human Exon 1.0 ST Array	Yes
GSE20291	Put	20	15	[HG-U133A] Affymetrix Human Genome U133A Array	No (no DEGs)

Data includes GEO Data Sets accession number, tissue, number of controls and PD patients, platform used and inclusion or exclusion criteria. The analysis and visualization of functional profiles was done with clusterProfiler [35]. Finally, we performed protein–protein interaction network analysis of the common differentially expressed genes (DEGs) with STRING, using only the highest confidence interaction score [36].

**Table 3:** Overlap between transcriptomic studies on the SN from PD patients.

	GSE7621	GSE42966	GSE49036	GSE20164	GSE54282	GSE20163	GSE20333	Our Data
DEGs	932	64	872	242	24	432	133	304
Overlap	223 (24%)	18 (28%)	229 (26%)	77 (32%)	2 (8%)	114 (26%)	19 (14%)	72 (24%)
Same direction	206 (92%)	14 (78%)	218 (95%)	71 (92%)	1 (50%)	103 (90%)	12 (63%)	66 (92%)

The table includes the accession numbers of the studies used, the number of differentially expressed genes with *p*-value < 0.01 and log2FC < −0.5849 or log2FC > −0.5849 (DEGs), the number of DEGs overlapping between all studies (overlap), and the number of overlapping DEGs that are either upregulated or downregulated in the same direction in all studies where they appear as DEGs. Percentages of overlapping genes from the total DEGs, and percentages of overlapping genes in the same direction from the total number of overlapping genes, can be found between brackets in the rows “Overlap” and “Same direction”, respectively.

**Table 4:** Overlap between microarray studies on the putamen from PD patients.

	GSE54282	GSE77666	GSE23290	Our Data
DEGs	56	4	2481	232
Overlap	7 (12%)	3 (75%)	35 (1.4%)	25 (11%)
Same direction	7 (100%)	1 (33%)	21 (60%)	13 (52%)

The table includes the accession numbers of the studies used, the number of differentially expressed genes with *p*-value < 0.01 and log2FC < −0.5849 or log2FC > 0.5849 (DEGs), the number of DEGs overlapping between all studies (overlap), and the number of overlapping DEGs that are either upregulated or downregulated in the same direction in all studies where they appear as DEGs. Percentages of overlapping genes from the total DEGs, and percentages of overlapping genes in the same direction from the total number of overlapping genes, can be found between brackets in the rows “Overlap” and “Same direction”, respectively.

**Table 5:** DEGs found both in the SN and putamen from PD patients and controls.

ENSEMBL	Gene Symbol	Protein Name	Protein Function
**Upregulated**			
ENSG00000089041	*P2RX7*	P2X purinoceptor 7	ATP receptor that acts as a ligand-gated ion channel
ENSG00000119699	*TGFB3*	Transforming growth factor beta-3 proprotein	Embryogenesis and cell differentiation
ENSG00000120729	*MYOT*	Myotilin	Myofibril assembly and stability
ENSG00000125551	*PLGLB2*	Plasminogen-like protein B2	Unknown
ENSG00000127530	*OR7C1*	Olfactory receptor 7C1	Odorant receptor
ENSG00000159842	*ABR*	Active breakpoint cluster region-related protein	GTPase-activating protein for RAC and CDC42
ENSG00000168209	*DDIT4*	DNA damage-inducible transcript 4 protein	Regulates cell growth, proliferation and survival in response to cellular energy levels and cellular stress
ENSG00000180015	*NA*		
ENSG00000186352	*ANKRD37*	Ankyrin repeat domain-containing protein 37	Unknown
ENSG00000188269	*OR7A5*	Olfactory receptor 7A5	Odorant receptor
ENSG00000214313	*AZGP1P1*	Pseudogene	
ENSG00000234769	*NA*		
ENSG00000244921	*NA*		
ENSG00000255823	*MTRNR2L8*	Humanin-like 8	Unknown
ENSG00000272755	*NA*		
**Downregulated**			
ENSG00000074317	*SNCB*	Beta-synuclein	Regulator of SNCA
ENSG00000078401	*EDN1*	Endothelin-1	Vasoconstrictor peptide
ENSG00000099957	*P2RX6*	P2X purinoceptor 6	ATP receptor that acts as a ligand-gated ion channel
ENSG00000103199	*ZNF500*	Zinc finger protein 500	*Transcriptional regulation*
ENSG00000137267	*TUBB2A*	Tubulin beta-2A chain	Major constituent of microtubules
ENSG00000148803	*FUOM*	Fucose mutarotase	Interconversion between alpha- and beta-L-fructose
ENSG00000155367	*PPM1J*	Protein phosphatase 1J	Serine/threonine protein phosphatase
ENSG00000164082	*GRM2*	Metabotropic glutamate receptor 2	Glutamate receptor
ENSG00000167306	*MYO5B*	Unconventional myosin-Vb	*Vesicular trafficking*
ENSG00000171532	*NEUROD2*	Neurogenic differentiation factor 2	Transcriptional regulation, implicated in neuronal determination
ENSG00000172794	*RAB37*	Ras-related protein Rab-37	GTPase that regulates vesicle trafficking
ENSG00000174807	*CD248*	Endosialin	*Angiogenesis*
ENSG00000185567	*AHNAK2*	Protein AHNAK2	*Calcium signaling*
ENSG00000196972	*SMIM10L2B*	Small integral membrane protein 10-like protein 2B	Unknown
ENSG00000198563	*DDX39B*	Spliceosome RNA helicase DDX39B	mRNA export from the nucleus to the cytoplasm; spliceosome assembly
ENSG00000234944	*LOC101929445*	Non protein coding—LINC02623	
ENSG00000272789	*NA*		
ENSG00000277400	*MAFIP*	MaFF-interacting protein	Coactivator of MAFF transcriptional activity

Up- and down-regulated DEGs found both in the SN and putamen samples, in the same direction. The table includes the ENSEMBL identification, gene symbol (when available), protein name (UniProt) and short summary of the protein function. Protein functions that are not certain are in italics.

**Table 6:** Statistical summary of the gene set enrichment analysis of the lipid metabolism genes in the SN and putamen from PD patients and controls.

Name	Source	NGenes	Direction	*p* Value	FDR	*p* Value.Mixed	FDR.Mixed
**SN**							
Phosphatidylcholines	GeneRIF Biological Term Annotations	5	Up	0.034	0.246	0.095	0.151
Phosphatidylcholine Biosynthesis	HumanCyc Pathways	6	Down	0.070	0.246	0.114	0.151
Phosphatidylcholine	Human Metabolome Database	81	Up	0.074	0.246	0.124	0.151
Phosphatidylcholines	CTD Gene-Chemical Interactions	36	Up	0.084	0.246	0.189	0.206
Phosphatidylcholines	dbGAP Gene-Trait Associations	3	Up	0.124	0.246	0.126	0.151
Phosphatidylcholine Biosynthesis Pathway	Biocarta Pathways	3	Down	0.134	0.246	0.056	0.151
GO:0031210	phosphatidylcholine binding	30	Up	0.144	0.246	0.111	0.151
GO:0046470	phosphatidylcholine metabolic process	14	Up	0.414	0.622	0.060	0.151
Phosphatidylcholinespecific	GeneRIF Biological Term Annotations	5	Up	0.693	0.866	0.538	0.538
GO:0034638	phosphatidylcholine catabolic process	5	Up	0.734	0.866	0.026	0.151
GO:0006656	phosphatidylcholine biosynthetic process	45	Down	0.794	0.866	0.025	0.151
Phosphatidylcholine	GeneRIF Biological Term Annotations	31	Down	0.949	0.949	0.123	0.151
GO:0008429	phosphatidylethanolamine binding	11	Up	0.096	0.390	0.254	0.355
Phosphatidylethanolamine	CTD Gene-Chemical Interactions	4	Up	0.130	0.390	0.480	0.480
Phosphatidylethanolamine	Human Metabolome Database	45	Up	0.344	0.687	0.272	0.355
GO:0006646	phosphatidylethanolamine biosynthetic process	15	Down	0.577	0.704	0.025	0.120
Phosphatidylethanolamine Biosynthesis	HumanCyc Pathways	5	Down	0.587	0.704	0.040	0.120
Phosphatidylethanolamine	GeneRIF Biological Term Annotations	13	Up	0.822	0.822	0.296	0.355
GO:0001786	phosphatidylserine binding	58	Up	0.046	0.457	0.064	0.214
Phosphatidylserines	CTD Gene-Chemical Interactions	9	Up	0.104	0.519	0.483	0.591
Phosphatidylserineexpressing	GeneRIF Biological Term Annotations	4	Up	0.308	0.955	0.532	0.591
Phosphatidylserine	DrugBank Drug Targets	10	Up	0.416	0.955	0.059	0.214
Phosphatidylserine	GeneRIF Biological Term Annotations	75	Up	0.611	0.955	0.202	0.337
GO:0006659	phosphatidylserine biosynthetic process	5	Down	0.752	0.955	0.125	0.256
GO:0006658	phosphatidylserine metabolic process	5	Down	0.777	0.955	0.514	0.591
Phosphatidylserine	Human Metabolome Database	45	Up	0.816	0.955	0.128	0.256
GO:0006660	phosphatidylserine catabolic process	8	Up	0.940	0.955	0.006	0.060
Phosphatidylserinebinding	GeneRIF Biological Term Annotations	3	Up	0.955	0.955	0.988	0.988
Phosphatidylinositolbinding	GeneRIF Biological Term Annotations	4	Up	0.032	0.380	0.521	0.556
Phosphatidylinositols	CTD Gene-Chemical Interactions	17	Up	0.134	0.493	0.052	0.304
Phosphatidylinositol4	GeneRIF Biological Term Annotations	3	Up	0.158	0.493	0.166	0.304
GO:0035091	phosphatidylinositol binding	99	Down	0.164	0.493	0.174	0.304
Phosphatidylinositol Signaling System	KEGG Pathways	72	Up	0.344	0.675	0.406	0.541
Phosphatidylinositol	GeneRIF Biological Term Annotations	331	Up	0.407	0.675	0.105	0.304
GO:0046488	phosphatidylinositol metabolic process	24	Up	0.479	0.675	0.203	0.304
GO:0006661	phosphatidylinositol biosynthetic process	78	Up	0.559	0.675	0.198	0.304
GO:0046854	phosphatidylinositol phosphorylation	53	Down	0.641	0.675	0.147	0.304
GO:0046856	phosphatidylinositol dephosphorylation	23	Up	0.648	0.675	0.557	0.557
Phosphatidylinositol3	GeneRIF Biological Term Annotations	24	Down	0.655	0.675	0.523	0.556
Phosphatidylinositol	Human Metabolome Database	85	Up	0.676	0.676	0.183	0.304
**Putamen**							
GO:0035091	phosphatidylinositol binding	99	Down	0.010	0.124	0.500	0.883
Phosphatidylinositols	CTD Gene-Chemical Interactions	17	Up	0.049	0.296	0.279	0.883
Phosphatidylinositol	Human Metabolome Database	85	Down	0.179	0.715	0.813	0.883
GO:0046854	phosphatidylinositol phosphorylation	53	Down	0.624	0.981	0.582	0.883
GO:0046856	phosphatidylinositol dephosphorylation	23	Down	0.723	0.981	0.883	0.883
GO:0046488	phosphatidylinositol metabolic process	24	Down	0.727	0.981	0.710	0.883
Phosphatidylinositolbinding	GeneRIF Biological Term Annotations	78	Down	0.748	0.981	0.726	0.883
GO:0006661	phosphatidylinositol biosynthetic process	4	Down	0.756	0.981	0.645	0.883
Phosphatidylinositol3	GeneRIF Biological Term Annotations	24	Down	0.759	0.981	0.749	0.883
Phosphatidylinositol	GeneRIF Biological Term Annotations	331	Down	0.919	0.981	0.495	0.883
Phosphatidylinositol4	GeneRIF Biological Term Annotations	3	Up	0.975	0.981	0.410	0.883
Phosphatidylinositol Signaling System	KEGG Pathways	72	Down	0.981	0.981	0.631	0.883
Sphingomyelin Metabolism/Ceramide Salvage	HumanCyc Pathways	8	Down	0.028	0.220	0.104	0.623
Sphingomyelins	dbGAP Gene-Trait Associations	3	Up	0.134	0.393	0.402	0.623
GO:0006685	sphingomyelin catabolic process	7	Down	0.147	0.393	0.167	0.623
Sphingomyelin	Human Metabolome Database	52	Down	0.232	0.464	0.764	0.764
Sphingomyelins	CTD Gene-Chemical Interactions	4	Down	0.334	0.535	0.467	0.623
GO:0006686	sphingomyelin biosynthetic process	6	Down	0.443	0.590	0.291	0.623
GO:0006684	sphingomyelin metabolic process	4	Down	0.556	0.635	0.716	0.764
Sphingomyelin	GeneRIF Biological Term Annotations	29	Up	0.900	0.900	0.339	0.623

Summary of results for the gene set enrichment tests of lipid metabolism genes. The table depicts the name of the pathway (Name), its source (Source) and the number of transcripts present in our dataset (NGenes). The results are shown with the controls (i.e., ‘Up’ refers to the gene set being increased in Parkinson’s disease (PD) patients compared to controls, and ‘Down’ the opposite). The hypothesis of ‘Mixed’ refers to the gene set including DEGs in both directions. FDR, false discovery rate; Mixed, non-directional analysis.

## Supplementary Materials

The following are available online at https://www.mdpi.com/2073-4409/9/9/1966/s1, Spreadsheet S1: Lipid analysis of SN and putamen from PD patients and controls; Spreadsheet S2: RNA-seq analysis of SN from PD patients and controls; Spreadsheet S3: Comparison between human SN transcriptomic studies; Spreadsheet S4: RNA-seq analysis of putamen from PD patients and controls; Spreadsheet S5: Overlap between the DEGs in SN and putamen from PD patients and controls.
